# Acute Kidney Injury Secondary to Hypertension-Related Thrombotic Microangiopathy: A Case Report and Literature Review

**DOI:** 10.7759/cureus.71067

**Published:** 2024-10-08

**Authors:** Achilleas Blatsos, Adel A Alalwan, Mohamed Razeem, Amanda Laird

**Affiliations:** 1 Department of Renal Medicine, Portsmouth Hospitals University National Health Service (NHS) Trust, Portsmouth, GBR

**Keywords:** acute kidney injury, ahus, atypical hemolytic uremic syndrome, malignant hypertension in ahus, thrombotic microangiopathy (tma)

## Abstract

The presence of acute kidney injuries (AKIs) in adults with microangiopathic hemolytic anemia and thrombocytopenia poses diagnostic and therapeutic challenges, as there are numerous causes that cannot always be rapidly differentiated. Treatment options vary widely, ranging from urgent treatments such as plasma exchange and anticomplement therapy to observation and supportive care. We report a case of AKI secondary to hypertension-related thrombotic microangiopathy and describe the clinical course from presentation to diagnosis and treatment. The patient remained hemodialysis-dependent despite attempts to control the blood pressure and administer anticomplement treatment.

## Introduction

A triad of thrombocytopenia, microangiopathic hemolytic anemia, and organ injury characterizes thrombotic microangiopathy (TMA). It has associations with a diverse range of medical conditions [1,2]. Malignant hypertension is an important cause of TMA that is likely to be unrecognized and underreported. It often presents a clinical conundrum in the initial assessment of acute TMA, as any patient with a TMA can conversely have severe hypertension [3]. It has an estimated incidence of 20%-40% in published case series from Nigeria and the Netherlands [4,5].

Acute kidney injury (AKI) is a common prominent feature of TMA because of the glomerular circulation propensity to endothelial damage and occlusion [2,6]. Early recognition of TMA in such cases is crucial due to associated morbidity and mortality, including end-stage renal disease, and the impact prompt initiation of supportive and specific treatments can have on outcomes.

## Case presentation

A 71-year-old woman, known to have diabetes mellitus, hypertension, iron deficiency anemia, dyslipidemia, and recurrent shingles, was referred by her general practitioner due to a new finding of raised serum creatinine on routine laboratory test (serum creatinine of 241 µmol/L at admission, from a baseline creatinine of 63 µmol/L three months prior). She also had a four-month history of exertional shortness of breath with less than ordinary activity and leg swelling. She had no history of chest pain or palpitations and did not report any changes in her bowel or urinary habits. She had a history of unintentional weight loss, with no fevers or night sweats, for which she had a contrast CT scan of her chest, abdomen, and pelvis before admission. The scan revealed bilateral symmetrical pleural effusions and no evidence of malignancy. Her regular medication regimen included metformin, sitagliptin, and atorvastatin.

Her blood pressure values on admission ranged between 170/80 and 190/90 mmHg, and her oxygen saturation was 96% on room air. On examination, she had bilateral basal crepitations, raised jugular venous pressure, and pitting edema up to the thighs. Her echocardiogram showed a left ventricular ejection fraction of 55%-60%, concentric left ventricular hypertrophy, and mild diastolic dysfunction. She also had a raised brain natriuretic peptide of 15,908 pg/mL (reference range <400 pg/mL) and a troponin T of 64 ng/L (reference range <12 ng/L). The rest of her laboratory parameters revealed an AKI stage 3 by serum creatinine criteria [7], worsening normocytic anemia, thrombocytopenia, and a raised lactate dehydrogenase with undetectable haptoglobin (Table 1). She also had evidence of hemolysis on the blood film with schistocytes (fragmented red blood cells) and a normal coagulation profile.

**Table 1:** Laboratory parameters during hospitalization: at admission, at one week (peak MAHA activity), and at three months of hospitalization ^a^The patient was hemodialysis-dependent from one week of hospitalization MAHA: microangiopathic hemolytic anemia

Laboratory parameter (reference range)	Results		
	At admission	At one week (peak MAHA activity)	At three months
Hemoglobin (120-150 g/L)	78 g/L	69 g/L	99 g/L
Platelet count (150-410 × 10^9^/L)	123 × 10^9^/L	57 × 10^9^/L	168 × 10^9^/L
Reticulocyte count (50-100 × 10^9^/L)	N/A	134.1 × 10^9^/L	88.32 × 10^9^/L
Creatinine (45-84 µmol/L)^a^	241	412	439
Urea (2.5-7.8 mmol/L)^a^	12.6	22.5	11.9
Lactate dehydrogenase (135-214 U/L)	456	653	227
Haptoglobin (0.3-2.0 g/L)	<0.10	<0.10	0.67

The working diagnosis was that of heart failure with preserved ejection fraction, uncontrolled hypertension, and AKI secondary to TMA. Given a platelet count of >50 x 10^9^/L and AKI stage 3 on presentation, the suspicion of thrombotic thrombocytopenic purpura as the cause of TMA was low. Therefore, therapeutic plasma exchange was not initiated while waiting for a disintegrin and metalloproteinase with a thrombospondin type 1 motif, member 13 (ADAMTS13) results. Initial medical management was focused on intravenous diuresis with furosemide and blood pressure control. A full workup was performed to investigate the TMA, including an infection screen (e.g., stool samples for Shiga toxin-producing *Escherichia coli*, STEC), autoimmune and vasculitis profiles, complement genetics, and complement antibody testing.

**Figure 1:**
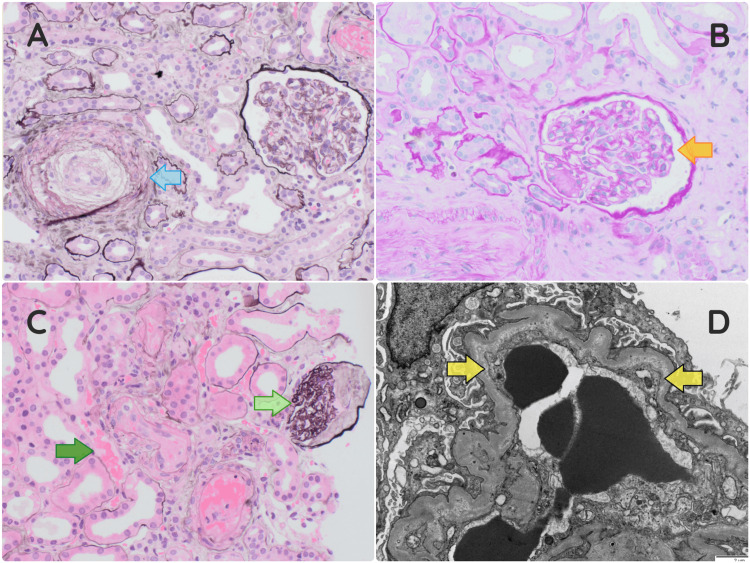
Native renal biopsy showing acute TMA predominantly affecting the arteries and arterioles. (A) Jones' stained section, original magnification 200×. It shows mucoid intimal thickening of an arteriole and an onion skin-like lesion (hyperplastic arteriolosclerosis) with a thrombus (blue arrow). (B) Periodic acid-Schiff-stained section, original magnification 200×. It shows a glomerulus with evidence of segmental mesangiolysis (orange arrow). (C) H&E-stained section, original magnification 200×. It shows a blood vessel with a thrombus (dark green arrow), an ischemic glomerulus with wrinkling of glomerular capillary walls (light green arrow), and a background of mild tubulointerstitial scarring. (D) Electron microscopy, original magnification 6000×. The glomeruli show focal subendothelial flocculent electron-lucent material (yellow arrows) Immunochemistry findings on renal biopsy: Congo red stain is negative. IgG: high background staining is present; there is focal and segmental capillary wall staining, which may be nonspecific trapping. Some of the damaged vessels show luminal and mural staining, and there is positive staining of what is possibly a detached small thrombus within the hilum of one glomerulus. IgA: no significant glomerular staining is seen, and there is weak staining of the damaged vessels, likely nonspecific trapping. IgM, C3, and C1q: an occasional glomerulus shows focal and segmental capillary wall staining of uncertain significance, and there is strong staining of the damaged vessels TMA: thrombotic microangiopathy; H&E: hematoxylin and eosin; IgG: immunoglobulin G; IgA: immunoglobulin A; IgM: immunoglobulin M

Regarding her workup, she had a positive (++) urinalysis for blood and protein with a urine protein-creatinine ratio of 316 mg/mmol (reference range <23 mg/mmol). Her STEC culture and polymerase chain reaction were negative. She had normal complement (C3 and C4) levels and a normal ADAMTS13 activity of 59.3 international units (IU)/dL (reference range, 60-146 IU/dL). She also had a negative autoimmune screen for antinuclear antibodies, anti-double-stranded DNA antibodies, anticentromere and anti-Scl-70 antibodies, antineutrophil cytoplasmic antibodies, antiglomerular basement membrane antibodies, anticardiolipin antibodies, and serum cryoglobulins. In addition, her serum protein electrophoresis did not reveal abnormalities with a normal serum-free light chain ratio. An ultrasound of her kidneys showed normal-sized kidneys (right kidney: 10.9 cm; left kidney: 11.4 cm) and normal echotexture. In addition, a renal biopsy was performed, which revealed acute TMA, predominately affecting the arteries and arterioles (Figures 1A-1D). Samples were also sent for genetic testing and factor H autoantibodies.

During the hospital stay, acute hemodialysis was initiated after one week due to worsening oliguria and fluid overload despite intravenous diuresis and attempts to control blood pressure. In addition, further expert advice was sought from the National Renal Complement Therapeutics Centre, and a three-month trial of eculizumab was instituted during the second week of hospitalization, given ongoing hemolysis and uncertainty of diagnosis, pending genetic and factor H autoantibody results. The protocol for the management of patients with atypical hemolytic uremic syndrome (aHUS) undergoing kidney transplantation was adopted for eculizumab dosing in this case [8].

Her admission was complicated by severe upper gastrointestinal bleeding. She had four endoscopies and, despite treatment, continued to bleed, requiring multiple blood transfusions and a subtotal gastrectomy. Following three months of treatment with eculizumab, she remained hemodialysis-dependent, and her complement genetics and factor H autoantibodies were negative (Tables 2, 3). Therefore, eculizumab was withdrawn as planned. Given the renal biopsy and the echocardiogram findings, the diagnosis was likely due to hypertension-related TMA rather than a primary complement-mediated event. This was further supported by the cessation of hemolysis at the end of the second month with tight control of blood pressure to a mean value below 140/90 mmHg. Future transplantation options were discussed in a multidisciplinary team meeting. If she were to be listed for a kidney transplant, she should not be given prophylactic eculizumab. However, further discussion is to be held in the event of further TMA episodes, as there would be no guarantee of response to treatment.

**Table 2:** Complement genetic testing results DNA from this patient has been analyzed by direct sequencing of the entire coding regions for the CFH, CFI, CD46, C3, CFB, DGKE, and MMACHC genes. In addition, MLPA analysis has also been carried out for deletions and duplications of the CFH, CFI, CD46, CFHR1, and CFHR3 exons CFH: complement factor H; CFI: complement factor I; CFB: complement factor B; DGKE: diacylglycerol kinase epsilon; MMACHC: metabolism of cobalamin-associated C; MLPA: multiplex ligation-dependent probe amplification; CFHR1: complement factor H-related 1; CFHR3: complement factor H-related 3

Genes tested	Results
CFH	No pathogenic variant detected
CFI	No pathogenic variant detected
CD46	No pathogenic variant detected
Complement C3	No pathogenic variant detected
CFB	No pathogenic variant detected
DGKE	No pathogenic variant detected
MMACHC	No pathogenic variant detected
CFHR1 (copy number)	1
CFHR3 (copy number)	1

**Table 3:** Complement screen and antifactor H antibodies results

Laboratory parameter	Results	Reference range
Complement C3	0.92 g/L	0.68-1.80 g/L
Complement C4	0.20 g/L	0.18-0.60 g/L
Complement factor H	0.46 g/L	>0.43 g/L
Complement factor I	27 mg/L	>21 mg/L
Classical hemolytic pathway activity (CH50)	89.41 U/mL	41.69-95.06 U/mL
Alternative hemolytic pathway activity (AP100)	Normal	
Antifactor H autoantibodies	Negative	

## Discussion

We reported a case of renal TMA with no identifiable genetic variants or antibodies, and a classic presentation of aHUS in the context of severe hypertension on presentation, defined as a systolic blood pressure of ≥180 mmHg or a diastolic blood pressure of ≥120 mmHg [9].

aHUS, described more recently as complement-mediated TMA, is a rare complement-mediated form of TMA that is relatively more common in children and young people than adults [10]. The incidence of complement-mediated aHUS in the United Kingdom is 0.42 cases per million per year [11]. The hallmark of aHUS is dysregulation of the alternative complement pathway with or without genetic abnormalities [12,13]. aHUS is largely considered a second-hit disease. Patients usually have preexisting genetic variations or risk alleles that predispose them to complement dysregulation, which can be triggered by severe hypertension, infections, pregnancy, solid organ transplant, certain drugs, malignancy, and other autoimmune conditions [1,14]. In almost one-third of patients, no triggering event has been identified [15]. Kidney biopsy cannot distinguish primary and secondary causes of aHUS. However, there are some clues to consider regarding clinical correlation. For instance, larger vessel (arterial and arteriolar) involvement has been shown to be associated with malignant hypertension [16].

Renal limiting TMA is more challenging to identify than multiorgan involvement, and delays are frequently encountered in determining the underlying cause. In addition, genetic testing and special antibody profiles can take days to weeks to process. This is a major concern, especially since these patients are often acutely unwell and have high rates of mortality without intervention. Thus, it is crucial not to delay the treatment when aHUS is suspected [1,2,5]. Treatment options for aHUS while awaiting genetic testing and antibody profiles include anti-C5 monoclonal antibody (eculizumab or eculizumab) and therapeutic plasma exchange, which should be considered in patients with a life-threatening disease or in places where this drug is not available [12,13].

TMA secondary to severe hypertension can occur by mechanical sheer stress on renal microcirculation (sheer stress-induced TMA) [3,10]. It can also be caused by a complement-mediated process [12,17]. In a cohort of 17 patients with hypertension-associated TMA, 14 patients had C5b9 formation on microvascular endothelial cells, and almost half of them (47%) had other rare variants in complement genes [17]. Treatment options in these cases can be affected by the presence of C5b9 formation and extrarenal involvement, such as neurologic disease, cardiac disease, hypertensive retinopathy, and systemic hemolysis [18]. Most patients with shear stress-induced TMA, normal C5b9 formation, and absence of extrarenal manifestations can be treated successfully with aggressive blood pressure control alone. Conversely, patients with strong C5b9 formation and extrarenal involvement (complement-mediated) have poor responses to blood pressure control, while complement inhibition appears promising [18].

Anticomplement therapy with eculizumab, as well as conservative management, has been described in the literature with variable success rates. Asif et al. described a case of aHUS with malignant hypertension as a presenting sign, marked AKI, and no identifiable complement gene mutations. The patient received eculizumab for one year, with normalization of hemoglobin and gradual improvement in renal function, resulting in stable chronic kidney disease [19]. Thind and Kailasam reported a similar case in which aggressive blood pressure control was obtained with multiple oral medications, and no additional special treatments were commenced. However, the patient's renal function did not improve, and he became dialysis-dependent [20]. In our case, the patient remained dialysis-dependent despite cessation of hemolysis with strict blood pressure control and failed to respond to three months of anticomplement therapy with eculizumab.

## Conclusions

Hypertension-related TMA is a rare but serious condition that can often lead to significant renal impairment requiring renal replacement therapy and carry high mortality without prompt intervention. TMA can be triggered by severe hypertension through direct endothelial damage and/or complement-mediated processes, and certain patients can have complement dysregulation from unidentified genetic variations or risk alleles. In our case, tight control of blood pressure resulted in the cessation of hemolysis. However, the patient failed to respond to three months of eculizumab treatment and remained hemodialysis dependent.
